# Women’s and clinicians perspectives of presentation with reduced fetal movements: a qualitative study

**DOI:** 10.1186/s12884-016-1074-x

**Published:** 2016-09-26

**Authors:** R. M. D. Smyth, W. Taylor, A. E. Heazell, C. Furber, M. Whitworth, T. Lavender

**Affiliations:** 1 Division of Nursing Midwifery and Social Work, University of Manchester, Manchester, M13 9PL UK; 2 Maternal and Fetal Health Research Centre 5th Floor (Research) St Mary’s Hospital Central Manchester NHS Foundation Trust, St Mary’s Hospital, Manchester, M13 9WL UK

**Keywords:** Reduced fetal movements, Qualitative, Interviews, Maternal experience, Clinicians, Management

## Abstract

**Background:**

Worldwide maternal perception of fetal movements has been used for many years to evaluate fetal wellbeing. It is intuitively regarded as an expression of fetal well-being as pregnancies in which women consistently report regular fetal movements have very low morbidity and mortality. Conversely, maternal perception of reduced fetal movements is associated with adverse pregnancy outcomes. We sought to gain insight into pregnant women’s and clinicians views and experiences of reduced movements.

**Method:**

We performed qualitative semi-structured interviews with pregnant women who experienced reduced fetal movements in their current pregnancy and health professionals who provide maternity care. Our aim was to develop a better understanding of events, facilitators and barriers to presentation with reduced fetal movements. Data analysis was conducted using framework analysis principles.

**Results:**

Twenty-one women and 10 clinicians were interviewed. The themes that emerged following the final coding were influences of social network, facilitators and barriers to presentation and the desire for normality.

**Conclusions:**

This study aids understanding about why women present with reduced movements and how they reach the decision to attend hospital. This should inform professionals’ views and practice, such that appreciating and addressing women’s concerns may reduce anxiety and make presentation with further reduced movements more likely, which is desirable as this group is at increased risk of adverse outcome. To address problems with information about normal and abnormal fetal movements, high-quality information is needed that is accessible to women and their families.

## Background

Worldwide maternal perception of fetal movements (FMs) has been used for many years to evaluate fetal wellbeing. It is intuitively regarded as an expression of fetal well-being [1] as pregnancies in which women consistently report regular FMs have very low morbidity and mortality [2, 3]. Conversely, maternal perception of reduced fetal movements (RFM) is associated with adverse pregnancy outcomes including: stillbirth [3, 4], fetal growth restriction (FGR) [3], preterm birth [5], oligohydramnios [6] and fetal abnormality [7]. A perception of RFM is frequently reported and occurs in approximately 5–16 % of pregnancies in the third trimester [8–10]. Although the absence of perceived fetal movements does not necessarily indicate fetal compromise or death [11] as many as 50 % of women perceive a gradual reduction in FMs several days before a stillbirth [10, 12, 13].

Given that RFM concerns so many women we propose that even a small improvement in women’s and clinicians’ understandings of RFM may have a substantial impact on antenatal care and pregnancy outcome. To use FMs as a screening tool for stillbirth prevention, the context in which these clinical presentations occur must be well understood. Therefore, we undertook a qualitative study of women and health professionals to further understanding of this situation. From the woman’s perspective we wanted to explore what triggers women to access health care after experiencing RFM and conversely what stops them. We interviewed clinicians to identify the practical challenges relating to the identification and management of women with RFM.

## Methods

A pragmatic interpretative approach was adopted, methods being driven by the research question [14]. A qualitative approach using the framework analysis method for data analysis [15] was used. Approval from the Research Ethics Committee (12/NW/0515) and Hospital Research & Governance Department was obtained. All participants gave written informed consent.

### Recruitment

Women attending a large teaching hospital in the North-West of England after experiencing RFM for the first time were invited for interview after clinical examination had excluded fetal death or fetal compromise. Clinicians at the study hospital did not routinely provide an information leaflet about RFM’s, although the handheld maternity records included a standard RFM paragraph. In addition, the hospital had a well-established (since 2011) RFM clinical protocol during the study time period. Midwives and obstetricians with experience of caring for women with RFM were recruited from the same hospital. Purposeful sampling permitted multiple perspectives to be captured [16]. Women were provided with information about the study at time of admission to hospital following RFM by the attending midwife or ultrasonographer. The clinical staff identified which women were eligible for the study and a study information sheet was provided at this point. Women were not required to decide either to join or decline participation to the interview at the time of admission. Rather, if they were willing, in principal, to take part they were approached the next day by WT to provide additional study information and arrange interview. Midwives and obstetricians were approached by WT and provided with a study information sheet. All participants were given a minimum of 24 h to consider participation.

### Data Collection

Data were generated from face-to-face audio-recorded semi-structured interviews that were transcribed verbatim; these were supplemented by field notes detailing relevant features of the interview. The interview schedules were designed to understand women’s and clinicians experiences and how they themselves make sense of RFM. Semi-structured questions included open-ended questions developed from expertise within the research team and published literature, thus gaining content validity [17]. This style of interview allowed the pre-specified topics to be explored in detail, as well as allowing exploration of new ideas mentioned.

The women’s interview schedule covered five broad topics; demographic details, general questions relating to the pregnancy, the women’s understanding and experience of fetal activity in pregnancy, information and advice provided by clinicians or others about RFM, and specific questions relating to their experience of RFM.

The interview for clinicians addressed four broad topics; demographic details, clinical monitoring of fetal activity, information they provided about RFM, and specific questions relating to the management of RFM.

### Analysis

Data were analysed using framework analysis principles - a flexible, rigorous and systematic method of analysis which entails the utilisation of a framework in five logical steps: familiarisation, identifying/developing a theoretical framework, indexing/charting and finally, mapping and interpretation [18]. The data from each group of participants were analysed separately and then merged together to explain the process of managing RFMs from women’s and clinicians perspectives. Rigour was maintained throughout the process by the research team conducting regular meetings to discuss the analysis of the data. To ensure anonymity and confidentiality each participant was assigned an individual number and all quotes anonymised. Individual codes denote two factors: interviewee number and length of time experiencing RFM before contacting a healthcare professional.

## Results

Of the 46 pregnant women approached, 21 (46 %) were interviewed; the remaining women could not be contacted to arrange an interview after initially indicating willingness to participate. At the point where no new or relevant insights were emerging from the data recruitment to the study ceased as it was considered data saturation was achieved [19]. Interviews with women took place from August 2012 to February 2013. One woman was interviewed in the postnatal period after giving birth earlier than expected; all other interviews were conducted antenatally. Women were between 20 and 40 weeks of pregnancy at the time of experiencing RFM and had waited between 6 h and 2 weeks after experiencing RFM before contacting a healthcare professional. Most interviews were performed at the women’s home (*n* = 18), the remainder at the study hospital. All clinicians were interviewed at their work place. Interviews lasted 24–60 min (mean time 39 min) for women and 12–34 min (mean time 23 min) for clinicians. There were no differences in terms of pregnancy characteristics between the women interviewed compared with those unable to be contacted (Table 1). Five midwives and five obstetricians were interviewed from the study hospital. One midwife who initially agreed to take part later declined as she did not wish to be audio recorded. Individual participant characteristics are shown in Table 2.

**Table 1:** Characteristics of women who were interviewed and those not interviewed

	Interviewed (*n* = 21)	Not interviewed (*n* = 25)
Primiparous	10 (48 %)	13 (52 %)
Gestation at time of RFM	32 weeks	35 weeks
Age (mean)	27 years	30 years
Ethnicity		
White British	13	15
Black British	1	6
Pakistani	3	2
Other	4	2
BMI (mean)	24	27^a^
Length of time experiencing RFM before contacting a healthcare professional (range)	6 h – 2 weeks	*6 h – 1 week* ^*b*^

^a^Missing data 1 woman

^b^Missing data 11 women

**Table 2:** Individual participant characteristics

Characteristics of Women						
Participant	Age	Parity	Gestation when experienced RFM	Ethnicity	BMI	Length of time experiencing RFM before contacting a healthcare professional
1	29	Primigravida	Term +1	White British	20	2 days
2	26	Primigravida	35 + 3	Pakistani	39	1 week
3	32	Multigravida	39 + 5	White British	26	6 h
4	22	Multigravida	20	White British	33	12 h
5	24	Primigravida	27	White British	20	3 days
6	26	Multigravida	30 + 6	White British	43	2 days
7	28	Multigravida	33 + 2	White British	30	1 day
8	36	Multigravida	36	Libyan	24	1 day
9	37	Primigravida	33	Italian	23	12 h
10	30	Multigravida	23	White British	31	2 days
11	29	Primigravida	Term +1	Black British	24	9 h
12	25	Primigravida	39	White British	26	2 days
13	21	Multigravida	39	Middle Eastern	31	8 h
14	28	Multigravida	33 + 4	White British	28	12 h
15	31	Primigravida	38	Mixed race	18	4 days
16	27	Primigravida	37 + 5	White British	24	1 day
17	34	Multigravida	34	White British	20	6 h
18	23	Multigravida	38	White British	25	2 weeks
19	33	Primigravida	34 + 3	Pakistani	22	3 days
20	22	Primigravida	34	Pakistani	29	2 days
21	31	Multigravida	35	White British	31	1 day
Characteristics of clinicians						
Participant	Age	Years’ experience in maternal health	Role			
22	41	12 years	Consultant Obstetrician			
23	42	17 years	Consultant in Obstetrics and Fetal Medicine			
24	38	14 years	Senior Lecturer and Honorary Consultant in Obstetrics			
25	36	11 years	Clinical Fellow in Obstetrics			
26	37	5 years	Clinical Fellow in Obstetrics			
27	52	26 years	Midwife/Sonographer			
28	40	5 years	Clinical Research Midwife			
29	50	17 years	Midwife/Sonographer			
30	29	7 years	Delivery Suite Midwife			
31	50	18 years	Delivery Suite Midwife			

### Findings common to all interviews

A finding common to all interviews was that women monitored their babies’ movements subconsciously: “*now, even a little bit while I’m talking he is kind of adjusting…. So he’s moving, I’m not looking for anything massive*” (12; 2 days). Fetal activity was universally perceived as an indication of the unborn baby’s good health, for example: *“you’re at peace with yourself and you know it’s fine” (20; 2 days).* Conversely, changes or a reduction in movements was non-reassuring: “*I was really worried because no movement means there’s something wrong with the baby*” (08; 1 day).

Notably, there was wide variation from one woman to another in how often their baby moved, making it problematic for individuals to specify the number of movements that would prompt concern; one woman discussed comparisons she made with other women: *“speaking to other people and hearing that their baby moved an awful lot, my baby might be absolutely fine, but she just doesn’t move much”* (04: 12 h)*.* Most women acknowledged there was a routine to their baby’s movements and sleep patterns. However, for some women their baby did not have a pattern making any assessment difficult for both women and clinicians: *“the problem was there didn’t feel like there was an obvious normal….I’ve had to really concentrate, like working out its routine”* (21; 1 day)*.*

From the analysis a number of major but related themes emerged. Themes were divided into three broad categories:

- Influences of social network
- Facilitators and barriers to seeking healthcare support
- Desire for pregnancy to be normal

### Influences of social network

#### People (Family, Peers)

When women were asked to specify sources of information on perception of fetal movements or fetal movement counting, the majority of women reported they frequently consulted family members or friends and prioritised views of their mothers, sisters or friends who were or had been pregnant over male partners’ views:

*“The first person I ring is my Mum” (18; 2 weeks)*

*“I think because he’s not a woman, so he wouldn’t know the experience of it, he’d probably give me his opinion, but he’d probably say, you know, ask your Mum” (13; 8 h)*

However, partners could validate the mothers’ concerns prompting engagement with maternity services:

*“My husband got me some pineapple juice; he goes ‘drink it fast’. It was ice cold; to shock the baby, wake it up. Did it and nothing happened and I started to panic” [11; 9 h]*

Some women were (falsely) reassured by family influences and therefore discouraged from attending hospital:

*“It was moving less, but I’ve always been told in our family by the time you get to the last month, there’s less space, so it’s going to move less, it made sense to me” (02; 1 week)*

For some, the consequence of ‘normalising’ the reduction of movements gave reassurance up until the point where no movements were felt:

*“… as long as she moved then I consider that to be okay. I think if it’s been a couple of days and they’ve not moved or a full day then it’s something to worry about” (18; 2 weeks)*

#### Internet (Websites, Forums)

Many women accessed information via a variety of parental web sites and forums although most interviewed here preferred to use trusted websites: *“I would Google, but I’d be more likely to go to NHS Direct”* (10; 2 days). Although some respondents found accessing forums helpful: *“I read forums, I don’t submit anything, I just like to read what other people have said, to see if any similarities from what I’m feeling, if it doesn’t then I ignore them”* (14; 12 h), others avoided the internet as they considered the information provided was either alarmist: *“When I searched them on-line I just felt myself getting terrified”* (20; 2 days) or presenting the *“worst case scenarios”* (03; 6 h).

From the interviews it was apparent that women frequently use the internet before or instead of consulting a health professional:

*“Yeah, just because it’s more accessible, you know, when you’ve got the internet on your phone and you’re thinking about something at nine o’clock in the evening, well, I’d rather go on the internet, because I don’t have my midwife’s number… so I’ll look on the internet to satisfy my own, sort of, curiosity.” (19; 3 days)*

### Facilitators and barriers to seeking healthcare support

#### Being taken seriously

From the current study it was evident that women’s unease with contacting the maternity services was linked to their perception that they would not be taken seriously by staff: *“I hate to feel like I’m wasting people’s time… like I’m being a hypochondriac.* (15; 4 days)*”* A goal of fetal movement monitoring is timely presentation to maternity services; however, a consequence of the perception of negative staff attitude meant that some women delayed reporting RFM:

*“And then eventually I just decided on the Sunday [48 h later] that it was time to go in, and you know it was lucky everything was alright, but in hindsight maybe I should have, maybe gone in a little earlier”* (12; 2 days)

This apprehension was compounded when on admission to hospital the same woman felt that her concerns were not taken seriously by clinicians:

*“I don’t feel that they (clinician) were really listening to me, they just made me feel daft for coming in” (12; 2 days)*

An account that was supported by some clinicians themselves:

*“I just take it (RFM) seriously; I think a lot of people don’t take it seriously. I think a lot of clinicians are quite blasé about it.” (24; obstetrician)*

#### Fear of something wrong with unborn baby

Our interviews explored what the triggers were for contacting the maternity services. Varying degrees of fear were prevalent prompting most to seek help:

*“Yes, just really worried that I was going to lose her. I just thought she was not happy or something wasn’t quite right. That’s obviously why I phoned because I just wanted to make sure she was alright.” (05; 3 days)*

Some women describing the heavy responsibility they felt:

*“It’s me that’s holding it, so that’s why I feel pressure. God forbid anything happened, there’s no-one else to blame for it if I’ve not monitored.”(07; 1 day)*

Although for some women their trigger to present to their maternity services was not initiated by their own concerns, but found inadvertently by the clinicians when asking about fetal movements at their next routine antenatal appointment:

*“it was the midwife when I saw her.….It had been 5 days to a week and straightaway she was like, you need to ring triage, we need to get it checked out. So that’s what prompted me to call in”* (02; 1 week).

Evident from our interviews was the prolonged length of time (up to 2 weeks) some women waited until they contacted a health professional. In some cases women’s denial of a problem, in the hope it would go away inhibited accessing healthcare:

*“I was a bit scared really, I didn’t really want to know was there something wrong, because that’ll be awful, because I’ve got this far, I’m nearly there” (12; 2 days)*

#### Fear of intervention

Some women actively delayed presentation to avoid intervention, particularly induction of labour or delivery: *“It’s like A and E, you don’t want to go to hospital if you don’t need to”* (05; 3 days)*, “… because obviously I don’t want to be induced or anything* (01; 2 days)*.”* However, clinicians’ perceptions were contrary to this:

*“This is where it does get a little bit grey. I think they know if they want to be seen about something else that they know if they say they’ve got reduced movements they’ll be taken seriously for complaints that they feel otherwise may be dismissed” (23; obstetrician)*

And as a consequence some women were less likely to contact services again:

*“I think I would be more nervous to go in if I felt reduced movements again, because I got the impression that they just thought I’d gone in to try and get induced or to try and persuade them to do something” (01; 2 days)*

### Mixed messages

There was a large variation and inconsistencies in the definition of ‘normal’ fetal movements, which was recognised to lead to confusion by both women and clinicians:

*“One of the problems I encounter is that there’s a wide broad-ranging experience and level of understanding of RFM which means that the information women are given varies greatly…. That’s why they’re confused, not because their family has misinformed them, but because they ask lots of different people and get lots of different answers.”(31; midwife)*

*“You hear different things, because somebody tells you every 12 h, somebody tells you a certain amount of movement doing all day. You question yourself, if gives me two kicks, one after another, do I count as one or two?” (09; 12 h)*

Almost all women reported being asked about fetal movements as part of their routine care, most of this advice was centred on the woman’s awareness of their baby’s pattern of movements: *“they’ve just said what’s felt normal for you* (10; 2 days)*”.* Some women wanted advice that was more tangible, wanting to know the time it takes to observe a specified number of movements: *“They haven’t told me how often or how many times she should move”* (06; 2 days)*.* As a consequence some women could not confidently recall at what point they should consider their movements to be suboptimal: *“I don’t know how bad it would have to be”* (20; 2 days) or considered the enquiry by professions as insufficient: *“I don’t think they’ve ever said what I should be feeling”* (06; 2 days)*.*

### Desire for pregnancy to be normal

#### Reluctance to present to maternity service

Of the women who recalled discussions with their clinician regarding fetal activity, some recalled receiving advice regarding what to do if they were concerned: *“If you don’t feel a movement it’s easy just come in”* (08; 1 day) *or “Are you happy with your baby’s movements…. if you are ever concerned then phone this number”* (17; 6 h). However, when we asked the women what they did once experiencing RFM there appeared a reluctance to contact the hospital straightaway. Instead, women tried to stimulate their baby with various means: exercise, cold drinks, sugar, chocolate, lying down:

*“We went for a walk around the park and I just thought he’d normally … he likes exercise, so it’s normally quite good. And then I thought I’d have a shower” (03: 6 h)*

*“I had some chocolate and some water and lay down” (16; 1 day)*

Some of this advice stemmed from professionals:

*“She (midwife/obstetrician) just said the information about lying down, drinking water or something sugary” (16: 1 day)*

Messages from staff when women came in with RFM were highly influential. Many women received reassurance: *“I left there feeling like I knew more, so just felt very well informed* (15: 4 days)*”* so consequently were confident to contact hospital again should they need*: “was very reassuring going out, that all I needed to do was pick up the phone if it happened again”* (09; 12 h)*.* Conversely, for some their experience was less positive: “*and the impact of their abruptness, it can be quite damaging, they just made me feel daft for coming in”* (12; 2 days).

#### Reassurance from investigations

There are a number of approaches to the management of RFM, of which women indicated that they appreciated staff using a CTG as way of providing them with the opportunity to focus on fetal activity in addition to hearing their baby’s heart beat *“It was the first time ever I’d actually sat and really actively considered whether baby was moving, was really reassuring”* (19; 3 days)*.* However, for some this only went some way of giving reassurance of normality *“Well when I heard it I was a lot more relaxed but I was still concerned as to why the hearts beating but, you know, she’s not moving”* (13; 8 h), another woman confirms she never doubted the baby was alive *“I just worried that there was something, like, I don’t know, there was something wrong*” (15: 4 days). A CTG in combination with an ultrasound scan provided some women with the additional reassurance they needed:

*“You get reassured, okay the baby’s alive, heart beating. But that doesn’t mean there’s nothing wrong with the limbs or something. I’d feel reassured if the sonographer could see that the limbs were fine, everything was fine” (20; 2 days)*

Some clinicians felt the same: “*we usually do a CTG which I think is good and reassuring for the women and the staff; then go in for scans-again it’s reassuring for the women.* (30; midwife)*”, “A scan is more reassuring because you’re looking at different parameters that a CTG is not going to tell you”* (25; obstetrician).

However, others grappled with wanting to reassure the women as quickly as possible using as little intervention as possible, but appreciating that some investigations by their very nature can be intrusive:

*“I think we probably over-manage it (RFM) because of technology and everything else, there’s no way out of that.” (28; midwife)*

## Discussion

**Fig. 1:**
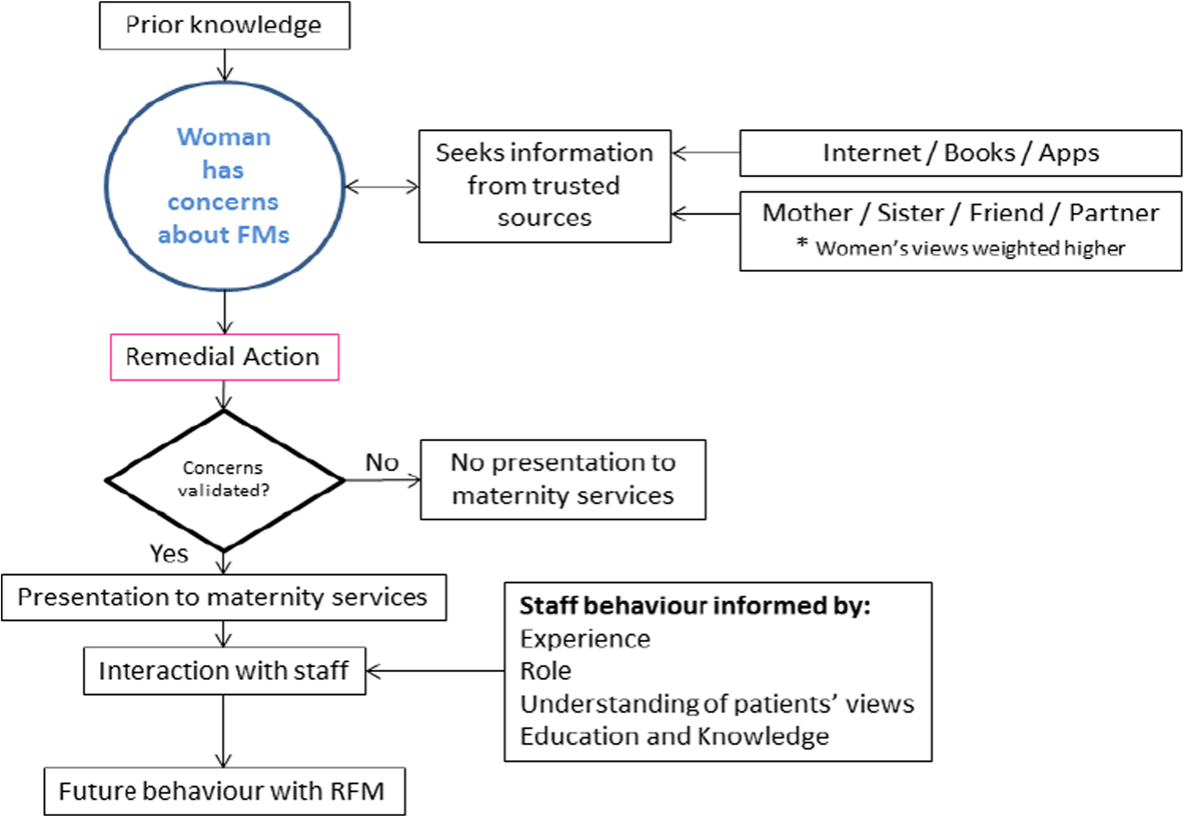
Conceptual model RFM

For the women interviewed here, their decision to present with RFM was influenced by their social network, by information about RFM obtained from various sources including the internet and maternity care providers, by concerns for their child’s wellbeing and a desire for a problem free pregnancy. Each of these individual components can be a facilitating factor or barrier to seeking healthcare support with RFM depending upon the individual context. It was clear that these women had a good knowledge of their babies’ pattern of movements. Following on from this observation, presentation with RFM was a considered process, during which women sought advice and closely monitored fetal activity. If mothers’ concerns were persistent or validated by external sources, they presented to their maternity service. Their interaction with care providers was usually affirming and provided much needed reassurance, however this was not always the case. Staff attitudes were critical in determining whether women would present again with perceived RFM (Fig. 1).

The main limitation of this study is that we interviewed women who presented to the maternity service with RFM; we were not able to identify and interview women who experienced RFM but did not present so cannot draw conclusions about barriers to presentation in this group. However, studies of women who have experienced stillbirth consistently report RFM prior to the diagnosis of fetal death [5] suggesting that future studies would need to include women whose babies were stillborn after RFM. This study was strengthened by a multidisciplinary approach that encompassed women, midwives and obstetricians. Although the group was diverse, reflecting the population of the maternity unit, the study would have been strengthened further by inclusion of multiple study sites.

Erlandsson et al. [20] explored women’s experiences prior to the diagnosis of stillbirth, focussing on reasons for presentation to maternity services. Their questionnaire-based study described that the majority of respondents had a premonition that their baby might be unwell, reporting a ‘feeling of unease’ and ‘not feeling the baby’ in the majority of cases. Although most women contacted their maternity care provider, a quarter of those who participated waited until their next antenatal care appointment, as was the case for some women with RFM in our study; thus information given to women needs to remind them to contact maternity services promptly with concerns, rather than waiting for a future appointment [21].

Irrespective of information and advice given to pregnant women, 6–15 % present with concerns about RFM in late pregnancy. Seeking health information or advice in pregnancy often reflects individuals’ circumstances in life, work and personal environment [22] but this need may only be triggered if it is preceded by knowledge to seek advice regarding fetal movements i.e. what is ‘normal’ for her baby. Midwives and obstetricians knowledge and practice regarding fetal movements and RFM is variable [23, 24] and guidance varies between units [25] Both staff and women had concerns about the mixed messages promoted by the internet, family-members and maternity staff. To address this, a consistent evidence-based message, regarding RFM, from maternity care providers must be delivered [21, 26], which enables the woman to seek help whilst taking care not to needlessly elevate anxiety.

The internet provides an immediate resource for health and pregnancy related concerns and, if used appropriately, has positive value but the information retrieved can be inconsistent and incorrect [27, 28]. Whilst it may prove invaluable to some women, for others it does not play a significant role, preferring to speak to a midwife as their first port of call [29]. Not only is it imperative that maternity care providers discuss current, evidence based information around fetal movements with women but also that they are able to guide women to reliable, high-quality web-based information [30]. Equally, advice from a woman’s social network may be inconsistent and inaccurate, which makes decision making a difficult task for the pregnant woman who receives conflicting advice from maternity care providers, internet, friends and family. If consistent advice is shared with pregnant women by maternity care providers, they will be able to recognise when advice from other sources is not valid and have confidence in themselves to seek help when necessary.

This study demonstrates women’s and professionals uncertainty about definitions of normal and abnormal fetal movements and its management highlighted by previous questionnaire studies [23, 24]. This may stem in part from the low-level of evidence underpinning the management of RFM. Two systematic reviews and meta-analyses of the management of RFM concluded that more studies were needed [11, 31]. Systematic reappraisal of existing data has moved the focus from counting specific numbers of movements to maternal awareness of fetal movements [32]. To address problems with information about normal and abnormal fetal movements high-quality information is needed that is accessible to women and their families.

## Conclusions

This study aids understanding about why women present with RFM and how they reach that decision. This should inform professionals’ practice, as appreciating and addressing womens’ concerns may reduce anxiety and make presentation with further RFM more likely, a desirable outcome as this group is at increased risk of adverse pregnancy outcome.
